# Feasibility of a 2^nd^ generation MR-compatible manipulator for transrectal prostate biopsy guidance

**DOI:** 10.1007/s00330-016-4504-2

**Published:** 2016-07-19

**Authors:** J. G. R. Bomers, D. G. H. Bosboom, G. H. Tigelaar, J. Sabisch, J. J. Fütterer, D. Yakar

**Affiliations:** 1 Department of Radiology, route 766, Radboud University Nijmegen Medical Center, P.O Box 9101, 6500HB Nijmegen, The Netherlands; 2 Soteria Medical, Arnhem, The Netherlands; 3 MIRA Institute for Biomedical Technology and Technical Medicine, University of Twente, Enschede, The Netherlands

**Keywords:** Prostate cancer, MRI, Biopsy, Robotics, Feasibility

## Abstract

**Objectives:**

To assess the feasibility of a 2^nd^ generation MR-compatible, remote-controlled manipulator (RCM) as an aid to perform MR-guided transrectal prostate biopsy in males with suspicion of prostate cancer (PCa).

**Methods:**

This prospective phase I study was approved by the local ethical committee and written informed consent was obtained from each patient. Twenty patients with ≥1 cancer suspicious region (CSR) with a PI-RADS score of ≥3 detected on the diagnostic multi-parametric MRI and no prior prostate treatment underwent MR-guided biopsy with the aid of the RCM. Complications were classified according to the modified Clavien system for reporting surgical complications. For evaluation of the workflow, procedure- and manipulation times were recorded.

**Results:**

All CSR’s (n=20) were reachable with the MR-compatible RCM and the cancer detection rate was 70 %. The median procedure time was 36:44 minutes (range, 23 – 61 minutes) and the median manipulation time for needle guide movement was 5:48 minutes (range, 1:15 – 18:35 minutes). Two Clavien grade 1 complications were reported.

**Conclusions:**

It is feasible and safe to perform transrectal MR-guided prostate biopsy using a MR-compatible RCM as an aid. It is a fast and efficient way to biopsy suspicious prostate lesions with a minimum number of biopsies per patient.

***Key Points*:**

*• It is feasible to perform transrectal prostate biopsy using a MR-compatible RCM.*

*• Using a RCM for MR-guided biopsy is safe, fast, and efficient.*

*• All cancer suspicious regions were reachable with the RCM.*

## Introduction

Men with a suspicion of prostate cancer (PCa), due to an elevated prostate specific antigen (PSA) and/or an anomalous digital rectal examination, undergo random systematic transrectal ultrasound (TRUS) guided biopsies to detect PCa. However, these biopsies are affected by underscoring and undersampling of PCa and have relatively low detection rates (22 – 42 %) [1–3].

Currently, multi-parametric MRI (mpMRI) is the most sensitive and specific imaging technique for localizing PCa [4]. Earlier reports indicated higher detection rates for clinically significant PCa with mpMRI and MR image-guided biopsies, which were previously missed with systematic TRUS-guided biopsies [5, 6]. Recently, it has been shown that MR image-guided biopsies resulted in fewer biopsies per patient compared with standard TRUS biopsy, and a decreased detection rate of clinically insignificant cancers [7, 8].

Nevertheless, there are some limitations associated with in-bore MR image-guided prostate biopsies. It can be time-consuming; site-experience with mpMRI and a trained prostate radiologist are needed. The majority of the procedure time in MR-guided prostate biopsy is lost during device manipulation. To facilitate correct alignment of the needle guide with the target lesion, the patient is repeatedly moved in and out of the MR scanner for device manipulation. To simplify and improve the process of needle placement, MR-compatible manipulators have been developed [9, 10].

The purpose of this phase I study was to assess the feasibility of a second generation MR-compatible manipulator as an aid to perform transrectal prostate biopsy in males with rising PSA and at least one suspicious lesion visible on the diagnostic mpMRI.

## Materials and methods

### Patients

This prospective phase I study was approved by the local ethical committee and written informed consent was obtained from each patient. Twenty consecutive patients with at least one cancer suspicious region (CSR) with a PI-RADS score of ≥3 detected on the diagnostic mpMRI, no prior treatment of the prostate, and scheduled for MR-guided biopsy were included. The most recent PI-RADS version was used [11, 12]. The mpMRI comprised T2-weighted, diffusion-weighted, and dynamic contrast-enhanced sequences and was acquired according the ESUR guidelines for prostate MRI [11]. The size of the CSR was measured on the ADC map. The maximal measured diameter was used to classify the CSRs in three different groups: CSRs ≤10 mm, CSR’s >10 mm and ≤20 mm and CSRs >20 mm. Exclusion criteria were contraindications to MR imaging (e.g., cardiac pacemakers, intracranial clips) and biopsy (e.g., rectal pathology).

### MR-compatible manipulator

**Fig. 1:**
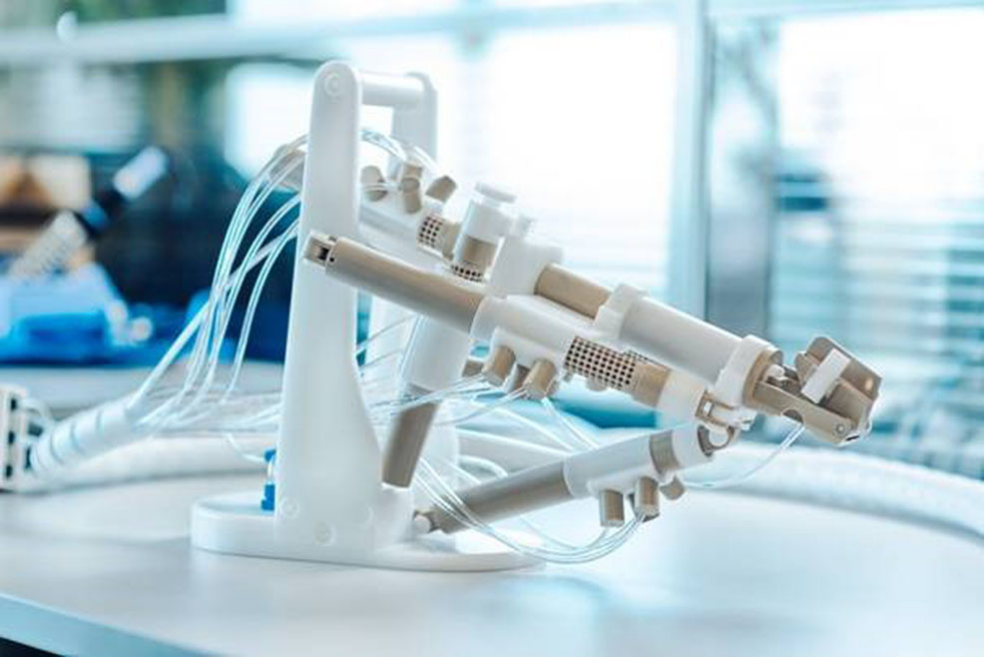
The remote controlled manipulator (Soteria Medical, Arnhem, the Netherlands)

The MR-compatible manipulator used in this study is a second generation remote controlled manipulator (RCM) (Fig. 1) developed by Soteria Medical BV (Arnhem, the Netherlands). The device is completely composed of plastic parts and tubing, preventing distortion of the magnetic field and enabling optimal patient safety. It is very compact, because it was designed to fit between the patient’s legs in the restricted space of the MR bore. The RCM is driven by pneumatic air stepper motors allowing fast and precise steps for the positioning of the needle guide. In combination with a stand-alone computer and dedicated interventional software for planning purposes and remote control, the manipulator positions the needle guide relative to the suspicious area by using the acquired images. This combination allows for a quick interaction to fine-tune the needle guide relative to the gland to adjust for either patient motion or tissue obstruction. The stand-alone computer and controller are located in the control room next to the MR console. The major differences between the new generation and the older version are the specifically designed stepper motors and the dedicated software. The new patented motor principle allows for a different geometrical setup and, therefore, a more compact design of the manipulator.

### MR-guided biopsy procedure

All patients received antibiotic prophylaxis (oral ciprofloxacin, two times a day 500 mg) during three days, starting on the day before biopsy. The MR-guided biopsy procedures were performed in a 3 T MR scanner (MAGNETOM Trio or Skyra, Siemens Healthcare, Erlangen, Germany) with the MR-compatible RCM.

**Fig. 2:**
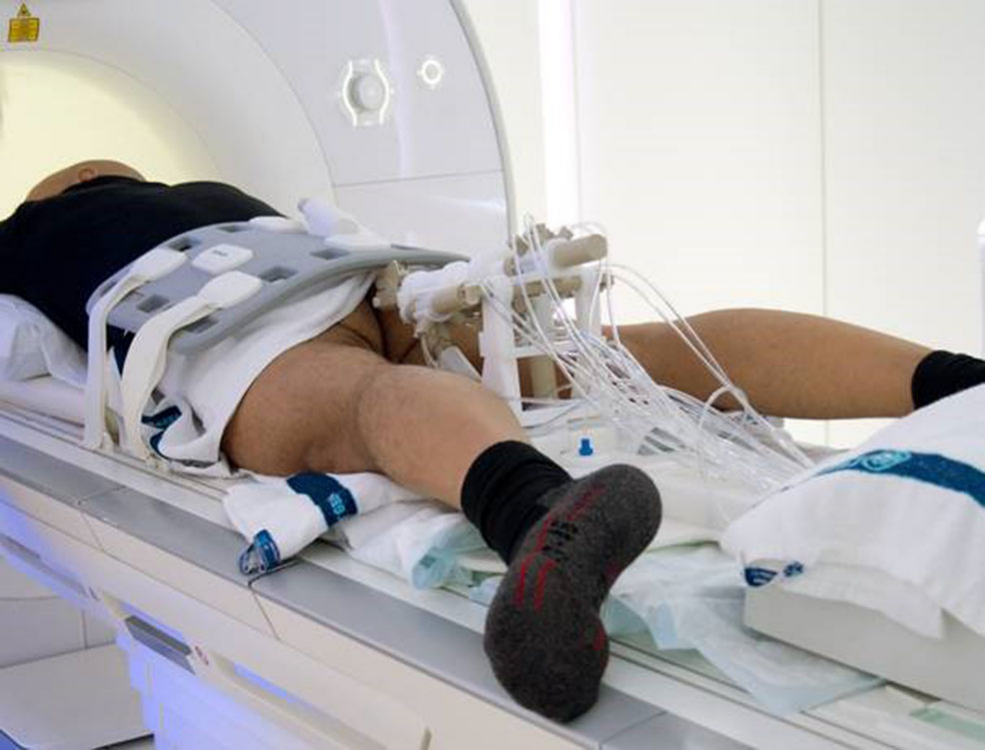
The remote controlled manipulator positioned between the patient’s legs

Patients were positioned in a prone position on the MR table with the RCM positioned between their legs. An MR-compatible needle guide (Invivo, Schwerin, Germany) was inserted in the rectum and attached to the RCM (Fig. 2). A body phased-array coil was placed over the patient’s pelvis for signal reception.

**Fig. 3:**
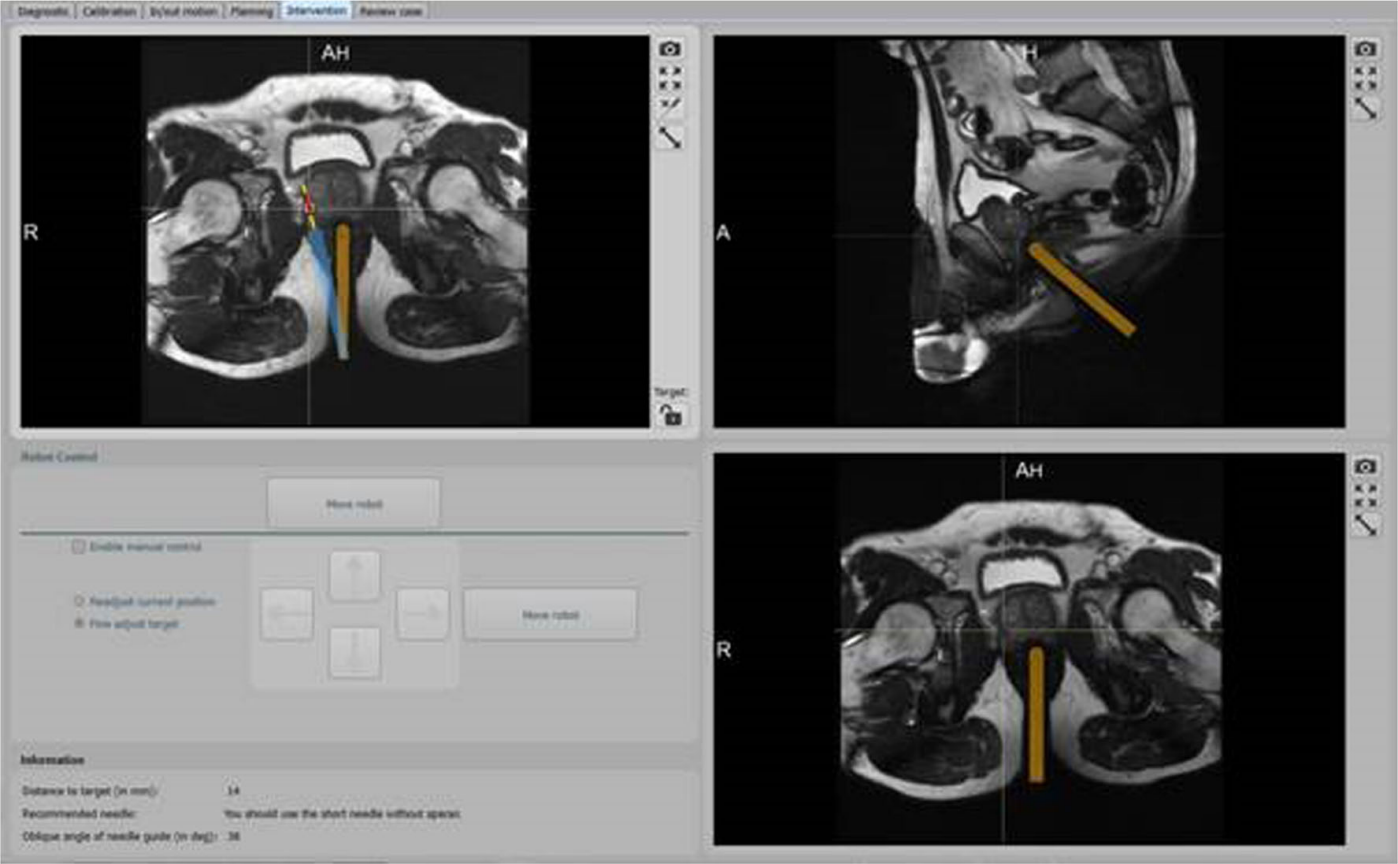
Screenshot of the planning software accompanying the remote controlled manipulator. The current position of the needle guide is represented by the orange line, the desired position for biopsy, the needle track and sample core are represented by the blue, yellow and red line, respectively

Axial T2-weighted - and diffusion weighted images (DWI) were acquired to relocalize the CSR previously detected on the diagnostic mpMRI. Directly after acquisition of the T2-weighted and DWI sequences, the images were sent to the standalone PC with dedicated interventional software, for optimal positioning of the needle guide for biopsy. The software automatically detected the needle guide and highlighted its position with a colour overlay on the MR images. If necessary, the rotation point of the needle guide could be adjusted manually. Subsequently, the radiologist planned and defined the desired target for biopsy. The software calculated the optimal path and the RCM steered the needle guide towards this area. The optimal position of the needle guide, the needle track and sample core were represented by a blue, yellow, and red line overlay (Fig. 3). For verification purposes of the new position of the needle guide, true fast imaging with steady state precession (TrueFISP) images in two directions was acquired.

If the needle guide position was not aligned correctly with the CSR, for example due to resistance of the anal sphincter or patient movement, the position of the needle guide was fine tuned with the help of the software followed by new TrueFISP images for verification. This step was repeated until the needle guide was correctly aligned with the CSR.

**Fig. 4:**
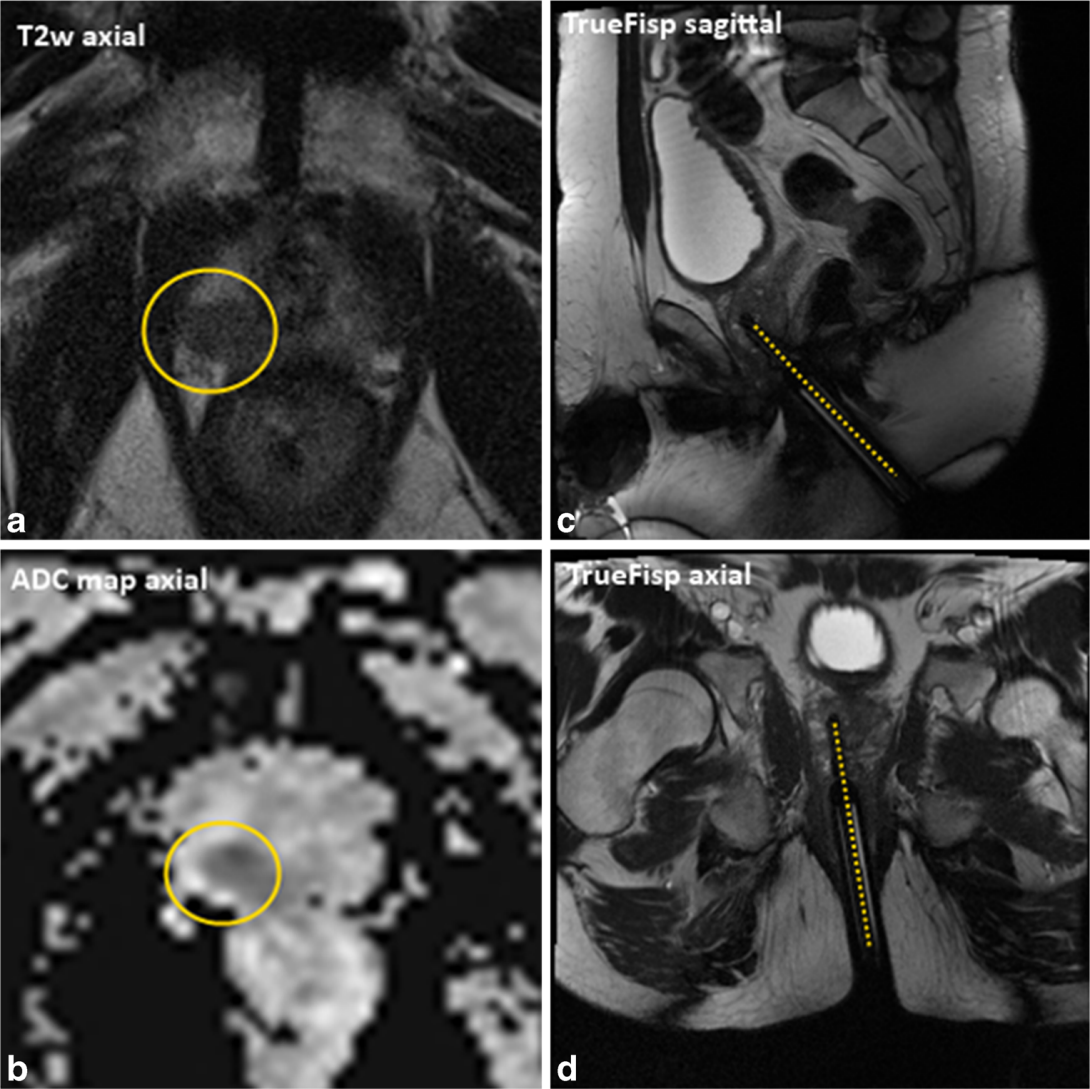
MR images of a 68-year-old patient, PSA 10.1 ng/mL and three negative TRUS biopsy sessions. **A+B:** MP-MRI showed a PI-RADS 5 lesion (yellow circle) in the right peripheral zone of the apex of the prostate. **C+D :** TrueFisp images in two directions acquired with the biopsy needle in situ. Histopathologic analyses showed a GS 3+4=7, cancer core length 7 mm

Eventually, after correct alignment of the needle guide with the CSR, a biopsy was taken. The position of the biopsy needle in situ was confirmed with TrueFISP images in the sagittal and axial planes (Fig. 4).

Feasibility and adverse events were reported with rates and description of adverse events. Surgical complications were classified according to the modified Clavien system for reporting surgical complications [13]. For evaluation of the workflow, procedure times were recorded. The total procedure time was defined as from the acquisition of the first localizer to the last confirmation image with the biopsy needle in situ. Manipulation time was determined as the time required for needle guide positioning towards a CSR. The set-up time of the RCM was not measured. The set-up of the RCM and its PC were quite straightforward; the RCM was positioned on the MR-table, and its PC was connected to the MR-computer. This was done simultaneously with patient positioning on the MR table and did not cost additional time.

### Histopathological analyses

The biopsy specimens were subsequently fixed in 10 % buffered formalin. Before being evaluated by a urogenital pathologist, the samples were embedded in paraffin, and stained with haematoxylin-eosin, according to our standard hospital protocol. All biopsy samples that were positive for PCa were assigned a Gleason score (GS) according to the Gleason-scoring system of 2005. Clinically significant PCa was defined as GS ≥ 3+4, or GS 3+3 with >1 positive core or a cancer core length > 6 mm [8].

## Results

In total, 20 patients were included in this study. All patient characteristics are shown in Table 1. All CSRs were reachable with the RCM and biopsy was performed in all patients.

**Table 1:** Patient characteristics

	Median (range)	
Age (y)	68 (51 – 76)	
PSA level (ng/mL)	11.0 (1.5 – 68)	
Previous negative TRUS-guided biopsies (#)	2 (0 – 5)	
Prostate volume (cc)	58 (25 – 272)	
Time between diagnostic multiparametric MRI and robot-assisted MR-guided biopsy (days)	30 (6 – 56)	
PI-RADS	PI-RADS 3	4
	PI-RADS 4	5
	PI-RADS 5	11
CSR size	≤10 mm	8
	>10 mm - ≤20 mm	7
	>20 mm	5
Biopsy outcomes	Normal tissue	1
	Prostatitis	4
	HGPIN	1
	GS 3+3	2
	GS 3+4	5
	GS 4+3	4
	GS 4+4	1
	GS 4+5	2
	Total	20

PSA = Prostate specific antigen; TRUS = Transrectal ultrasound; MRI = Magnetic Resonance Imaging; PI-RADS = Prostate imaging and reporting archiving data system; CSR = Cancer suspicious region; HGPIN = High-grade prostatic intraepithelial neoplasia; GS = Gleason Score

Two minor complications, classified as Clavien Grade 1, were reported. One man experienced extreme nausea and felt the urge to move after sampling the second biopsy core. This led to activation of the safety mechanism, which detached the needle guide from the RCM. Two additional biopsy cores were sampled manually. Another patient fainted directly after the MR-guided biopsy procedure.

### Procedure time

The median procedure time was 36:44 min with a range of 23 – 61 min. Median manipulation time for needle guide movement was 5:48 min (range, 1:15 – 18:35 min). No additional time was needed to set-up the RCM.

### Histopathologic outcomes

A total of 20 prostate lesions with a PI-RADS score of 3 or higher were detected in 20 patients. A median of two biopsies per lesion (range, 2 – 4) were taken. Fourteen out of 20 lesions (70 %) were proven to be PCa. Twelve out of 20 (60 %) were clinically significant. Four biopsies contained prostatitis, one contained high-grade prostatic intraepithelial neoplasia (HGPIN) and one contained normal prostate tissue. Eleven of the lesions were located in the peripheral zone and nine were located in the transition zone.

## Discussion

MR-guided transrectal prostate biopsy with the aid of an MR-compatible RCM was a feasible and safe procedure. All CSRs were reachable with the RCM and the cancer detection rate was 70 %. The median procedure time was 36:44 min (range, 23 – 61 min) and our median manipulation time for needle guide movement was 5:48 min (range, 1:15 – 18:35 min).

Yakar et al. described the results of the first generation MR compatible manipulator and since then no other studies describing this procedure have been reported [9]. They reported a median biopsy procedure time of 76.5 min (range, 45 – 105 min), a median manipulation time of 2.5 min (range, 1 – 5 min) and a cancer detection rate of 56 %. A systematic review on transrectal MR-guided prostate biopsies, published in 2013 by Overduin et al., found nine papers describing this procedure in a closed bore system [14]. In these nine papers, 684 patients were biopsied with a median cancer detection rate of 42 % (range, 8 – 59 %), and the median procedure time for MR-guided biopsy in a closed bore setting was 55 min (range, 30 – 68 min).

Compared to our data, the main improvement is the major decrease in total procedure time. With the current RCM, the median procedures times were approximately 40 min and 20 min faster than with, respectively, the first generation manipulator as with the standard manual procedure. The major goal of the development of the RCM was to speed up the biopsy procedure and our results showed that this is possible. However, our results might have been biased because per patient only one CSR was biopsied. Despite the decrease in total procedure time, the median manipulation time with the RCM was longer than the median manipulation time of the first-generation manipulator.

Possible explanations for this are the additional time needed to transfer the data, the repeated learning curve for multiple users as well as the built-in safety mechanism of the RCM. When the needle guide experienced resistance during movement, the RCM stopped moving. This sometimes led to extra iterations of needle guide movement, especially for CSRs located in the base or apex of the prostate or CSRs located very laterally. An upgrade of the RCM software enabling adjustment of the rotation point of the needle guide is expected to improve this.

Next to this, our cancer detection rate was higher than described in the other studies. Multiple factors may account for this, as for example the high number of PI-RADS 5 lesions (11/20), or it may indicate that performing targeted prostate biopsy with our RCM is accurate. However, the real biopsy accuracy should be examined in an additional study.

Another emerging way to perform targeted prostate biopsies is with MRI-TRUS fusion. Previously acquired MR images are fused with real-time TRUS images to acquire targeted tissue samples from the suspicious area. Major advantages of this technique are that no expensive MR scan time and MR-compatible materials are needed when performing a biopsy, however it does require significant investment in additional equipment. A recently published review on this subject found a median cancer detection rate of 50.5 % (range, 23.7 – 82.1) in a total of 2293 patients [15]. Procedure times were not reported. This was done by Brock et al., who performed two targeted fusion guided biopsies in 52 patients and found a mean biopsy procedure time of 15 min (range, 8 – 49 mins); however their detection rate was only 26.4 % [16]. Comparison with manual MR-guided biopsy is difficult, because in most papers more than one lesion is targeted and the time per lesion is barely specified. In theory, it may turn out that MRI-TRUS fusion biopsy is especially suitable for patients with large, high-grade PCa lesions, because these are often better visible on ultrasound. In our opinion, in-bore MR-guided biopsy is more preferable for the smaller lesions. However, direct comparison with prospective randomized controlled trials between MRI-TRUS fusion biopsy and in-bore MR-guided biopsy are needed to find out which technique is the most appropriate to detect PCa. Recently, Arsov et al. published the results of a randomized study investigating the detection rate of MR-guided in bore biopsy versus the combined approach of MRI-TRUS fusion biopsy with systematic TRUS biopsy [17]. No significant difference was found between both groups.

Our study had a number of limitations. The RCM and its software were continuously optimized during this study. However, this is inherent to the natural process of a feasibility study. The most significant adjustments were the introduction of a direct connection between the MR console and the standalone RCM PC to send MR images, and the introduction of the software option to adjust the rotation point of the needle guide. It is expected that these adjustments will even lead to a further decrease of the manipulation and procedure time. Another limitation of this study was the relatively small patient population.

The next step is to examine the biopsy accuracy of the RCM. For further studies, the application of the RCM in combination with an automated real-time needle-guide tracking sequence [18], or during MR-guided transrectal targeted therapies, as for instance focal laser ablation, can be investigated [19]. With some minor adjustments, the RCM may also be used during transperineal prostate biopsies and targeted therapies, as for example MR-guided cryoablation [20].

## Conclusion

It is feasible to perform transrectal MR-guided prostate biopsy using a remote controlled, MR-compatible, manipulator as an aid. It is a fast, and, therefore, efficient way to biopsy suspicious prostate lesions with a minimum number of biopsies per patient.

## Abbreviations

CSR: Cancer suspicious region
DWI: Diffusion weighted imaging
GS: Gleason Score
HGPIN: High-grade prostatic intraepithelial neoplasia
mpMRI: Multi-parametric magnetic resonance imaging
MR: Magnetic resonance
PCa: Prostate cancer
PI-RADS: Prostate imaging and reporting archiving data system
PSA: Prostate specific antigen
RCM: Remote controlled manipulator
TrueFISP: True fast imaging with steady state precession
TRUS: Transrectal ultrasound
